# Laceration of the Perineum

**Published:** 1887-11

**Authors:** Ben. H. Brodnax

**Affiliations:** Brodnax, La.


### LACERATION OF THE PERINEUM.

*To the Atlanta Medical and Surgical Journal:*

I notice, on page 403 of the September number of your JOURNAL, an appeal by Dr. J. M. Sanders, of Magnolia, S. C., to several of the arts to invent some mode whereby greater capacity (elasticity) may be given to the perineum to prevent laceration.

I am not a professor of any of the specified arts, but trust that a little experience of my own will be of use to him—perhaps others.

I have attended six cases of primipara over 30 years old—one 41 years of age. My first case, aged 32, I had the misfortune to have a lacerated perineum. In the five others, having burned my fingers with the first, I was more cautious, acting on the idea that to a certain extent the combination of muscles that go to form vulva and perineum are a sphynctus, I commence sometime (an hour or so) before the head had descended against the perineum to apply to the vulva a cloth wrung out in a hot solution made as follows: Water as hot as can be borne, one pint; fluid extract belladonna, four ounces; mix. With the right hand, this was kept pressed to the parts and renewed as it cooled. As the head comes down I arrange it to accommodate the face-occiput to the long diameter of pelvis, and it passed out readily.

Then, taking the head between both hands, I steadily raised the ear towards the groin of the mother, when the shoulder opposite slipped out. Inserting a finger, pressed the arm across the breast and pulled it out. Then, reversing the movement of the head, pressed the left ear toward the buttock of the mother's opposite side, and the other shoulder came out; second arm removed same as first one.

I have had but the one case of perineum laceration, and the manipulation above described is so simple that any cool-headed doctor can carry it out.

Dr. Sanders is, of course, aware that the head is not always the cause of the laceration, the greater number being caused by the sharp shoulder process (acromion) pressing on the thinned muscles. And right here let me give you my other aid. It is a well-known fact that kneading the womb, or even steady pressure with the palm of the hand, will increase and make the pains more steady, and the head having passed out, the physician needs both hands to manipulate as above. If, while the pains are coming on, but the womb seems not able to make the child advance, he will take a large towel, fold it diagonally to six inches wide, pass one end under the back of patient, draw it out on the right side, bring the other end over the abdomen, place it so that the lower edge will come down to the navel. Take a half turn with each end on the other (hook them together as you would two fingers of opposite hands) hand one end to attendant on woman's left side, and retain the other in left hand. By tightening the bandage after each pain,he will assist the womb in holding what it has gained at each effort, and will find in difficult labors that the duration is very materially shortened.

Then, when the head of child has protruded, he hands his end to an assistant on the woman's right side, instructing her how to tighten and hold it, and has both hands free to manage the head of the child.

I have used both the above methods for the last eighteen or twenty years and find them satisfactory in every respect.

If any other bandage than the towel is wished, a yard of white goods folded six inches wide, a stick sewed into each end to keep it extended, with a *ring and strap sewed on each end*, so that it can be tightened from either side of the woman, costs about twenty cents.

I am not sure but the application of the belladonna may lull pain at the last act of birth—at least I find very little use for chloroform and very little complaint from patients.

DR. BEN. H. BRODNAX.

*Brodnax, La., Sept. 30th, 1887.*

